# The effect of hepatic steatosis on 18F-FDG uptake in PET-CT examinations of cancer Egyptian patients

**DOI:** 10.1186/s41824-023-00173-6

**Published:** 2023-10-16

**Authors:** Magdi A. Ali, Eman El-Abd, Mohamed Morsi, Mohamed M. El Safwany, Mohamed Z. El-Sayed

**Affiliations:** 1 Medical Research Institute, Alexandria University, Alexandria, Egypt; 2 Faculty of Applied Health Science Technology, Pharos University in Alexandria, Alexandria, Egypt; 3 Faculty of Health Sciences, Gulf Medical University, Ajman, United Arab Emirates

**Keywords:** ^18^F-FDG PET-CT, Body mass index, Liver steatosis, SUVmax

## Abstract

**Background:**

Hepatic steatosis is the most common chronic hepatic disease. Imaging diagnosis of hepatic steatosis has been evaluated as an alternative to invasive histological diagnosis.

**Study aims:**

The study aimed to assess the effect of hepatic steatosis on Flourine-18 fluorodeoxyglucose (18F-FDG) uptakes in cancer patients.

**Patients and Methods:**

Blood samples were collected from 50 cancer patients and analyzed to calculate fatty liver index and Hepatic steatosis index (HIS). Hepatic steatosis examined using high-resolution ultrasound and positron emission tomography—computed tomography (PET-CT). Linear attenuation coefficient, standardized-uptake value (SUV) mean (SUV mean), and SUV maximum (SUVmax) were measured. Accordingly, patients were divided equally into non-fatty liver, and fatty liver groups.

**Results:**

A significant increase in SUVmax and SUV mean was observed in the fatty liver group more than in the non-fatty liver group. HSI significantly increased in the fatty liver group compared to the non-fatty liver group. Liver tissue uptake FDG was significantly correlated with HSI values. SUV max significantly correlated with body mass index (BMI) in the non-fatty group only.

**Conclusion:**

Hepatic changes in cancer patients affect the liver metabolic activity and thus the 18 F-FDG uptake. Therefore, further corrections should be considered when the liver is used as a comparator for PET-CT scans of cancer patients.

## Introduction

Fatty liver disease reflects a wide spectrum of conditions characterized histologically by excessive accumulation of triglycerides and cholesterols within the cytoplasm of > 5% of hepatocytes (Sanyal et al. 2011). Hepatic steatosis is caused by an abnormal and excessive intracellular accumulation of fat (mostly triglycerides) in hepatocytes. It is a common radiologic finding. Fat accumulation in the liver occurs in six patterns: diffuse, regional, localized, subcapsular, multifocal, and perivascular (Hu et al. 2022).

Fatty infiltration of the liver is further subdivided into alcoholic fatty liver disease and non-alcoholic fatty liver disease (NAFLD) (Özülker and Özülker 2019).

NAFLD or MAFLD (metabolic associated fatty liver disease) includes two pathological entities; simple steatosis and non-alcoholic steatohepatitis (NASH) (Salomon et al. 2018).

NAFLD is a complex disease that involves multiple organs and diverse mechanisms and results from the interplay between metabolic and environmental factors with genetic and epigenetic predispositions (Juanola et al. 2021).

In a small number of cases NAFLD develop into NASH and progress towards end stage liver diseases (Keramida et al. 2020).

In Egypt, NAFLD “remains unknown due to the lack of large population-based studies” (Alboraie et al. 2019). Liver biopsy is the gold standard to differentiate NASH from simple steatosis and identify the advanced hepatic fibrosis but it is invasive, poorly acceptable, expensive, and has sampling variability (Castera et al. 2019).

Noninvasive techniques include quantification of serum biomarkers and measurement of liver stiffness, using either ultrasound- or magnetic resonance-based elastography are investigated (Castera et al. 2019).

Algorithms based on serum biomarkers such as Fatty Liver Index (FLI) and Hepatic Steatosis Index (HSI) were extensively used. FLI value varies between 0 and 100 (A FLI < 30 rules out and a FLI ≥ 60 rules in fatty liver) and was used as an accurate predictor for fatty liver in the general population (Bedogni et al. 2006). HSI is an efficient simple index based on standard laboratory tests and anthropometric parameters used as a screening tool for NAFLD (an HSI < 30.0 rules out and an HSI > 36.0 rules in NAFLD) (Lee et al. 2010).

The diagnostic accuracy of serum biomarkers is suggested to be improved by combining them with different approaches such as imaging modalities (Castera et al. 2019).

Conventional imaging techniques [Ultrasonography (US), computed tomography (CT), and magnetic resonance imaging (MRI)] have low to moderate accuracy to identify liver fibrosis (Allan et al. 2010; Lo et al. 2017). Sonography and unenhanced CT effectively detect steatosis if fatty infiltration is > 10% and > 30%; respectively (Obika and Noguchi 2012; Ballestri et al. 2017; Hajong et al. 2018). Fluorodeoxyglucose positron emission tomography/CT (FDG-PET/CT) plays a specific role in assessing diagnosis, staging, and monitoring therapeutic response in oncology imaging (Luk et al. 2013).

The standardized uptake values (SUVs) are used to eliminate the variability resulting from differences in patient size and the amount of the injected FDG (Lin et al. 2011).

Then the mean value within a fixed size of a region of interest (ROI), SUV maximum (SUVmax), the use of “reference tissue” SUVmax values and normalization of lesion/target SUV measures to those of selected reference tissues were introduced to reduce variability of SUV measurements (Fletcher and Kinahan 2010). Liver was among the tissues that have been advocated as reference tissue and showed the least inter-patient coefficient of variance (0.21) (Fletcher and Kinahan 2010).

Several studies investigated the possible effect of fatty infiltration on liver SUVs (Abikhzer et al. 2011; Kim et al. 2011; Keramida et al. 2014; Keramida et al. 2014; Liu et al. 2015; McDermott et al. 2019; Rozenblum et al. 2020; Seraj et al. 2019).

Frequently, liver FDG uptake is used as the benchmark for diagnosis, treatment assessment, prognosis, and quality control in PET/CT imaging. A number of factors, including age, blood sugar, body mass index (BMI), incubation time, and hepatic steatosis, affect the liver’s capacity to absorb FDG. There are several factors that could affect the SUV-measured FDG uptake, including weight, plasma glucose level, interval length, partial volume effects, and recovery factor. It has been demonstrated that overweight or obesity and non-alcoholic fatty liver disease are linked, and that the metabolic syndrome is characterized by alterations in normal glucose metabolism (Ahmed et al. 2022). However, the relation between fatty infiltration of the liver and FDG uptake in terms of SUVmax and SUV mean values remains unresolved. Therefore, the current study was designed to assess the effect of hepatic steatosis on 18F-FDG uptake in PET-CT examinations and to evaluate the efficiency of using the liver as an internal reference organ in the Egyptian cancer patients.

### Patients and methods

The current study included 50 subjects (with no definite focal fatty changes or focal fatty sparing areas) from those who refereed to perform PET-CT examination in Ayadi Al-Mostakbal Oncology Center (Alexandria, Egypt) from 1st of June 2020 to 20th of December 2020. Subjects were recruited according to the rules of Ayadi Al-Mostakbal ethical committee for conduction of medical research on human subjects and informed consents “were obtained from each patient included in the study.

The study protocol conforms to the ethical guidelines of the 1975 Declaration of Helsinki (6th revision, 2008) as reflected in a priori approval by the institution’s human research committee”. Other liver diseases, such as alcoholic liver diseases, and viral liver diseases are excluded. Deoxygenated blood sample were collected from patients before injection of the 18F-FDG to analyze the alanine amino transferase (ALT), aspartate amino transferase (AST), gamma-glutamyl transferase (GGT), fasting plasma glucose (FPG), triglycerides (TGs) which were measured using fully automated (Roche, Cobas c 311 analyzer, India) and values were used to calculate FLI and HSI.

Hepatic steatosis patients were identified by using High Resolution US (HRUS; General Electric model LOGIQ S7, USA, C1-5-D broad- spectrum convex transducer with field of view 70° and frequency range 1.8-5 MHz) and non-contrast computed tomography (NCCT). Patients were scanned separately on an integrated PET- CT scanner with 2D image acquisition after injection of 18F-FDG (without contrast) (Siemens Biograph 64-slice PET scanner, Germany, 120 KVp/50 mA-Care dose; slice thickness 5 mm; pitch 0.8; rotational speed 0.5/sec, convolution kernel B19f PET, very smooth) then Digital Imaging and Communications in Medicine (DICOM) images were extracted from PET-CT scan and sent to workstation. Both of the attenuation coefficient value of the liver and spleen tissues were measured using region of interest (ROI) avoiding any lesions, biliary, vascular and artifacts. SUV mean, and SUVmax of the liver were also measured on PET scan. Accordingly, patients were subdivided into two groups:

1. Control group (25 patients) with a mean liver attenuation value ≥ the mean spleen attenuation value.
2. Diffuse fatty liver (hepatic steatosis) group (25 patients) with a mean liver attenuation value < the mean spleen attenuation value.

## Results

US revealed absence of fatty liver in 50% (25/50) of patients and variable grades of fatty liver status in 50% (25/50) of patients [grade 1 (mild): 13 (26%); grade 2 (moderate): 8 (16%); and grade 3 (sever); 4 (8%)].

Based on the CT the HU liver to spleen ratio was < 1 in 25 patients (11 of grade zero, 9 grade 1, 3 grade 2, and 2 grade 3 as detected by US) designated as fatty liver group. While, HU liver to spleen ratio was ≥ 1 in 25 patients (14 of grade zero, 4 grade 1, 5 grade 2, and 2 grade 3 as detected by US) designated as non-fatty liver group.

**Fig. 1:**
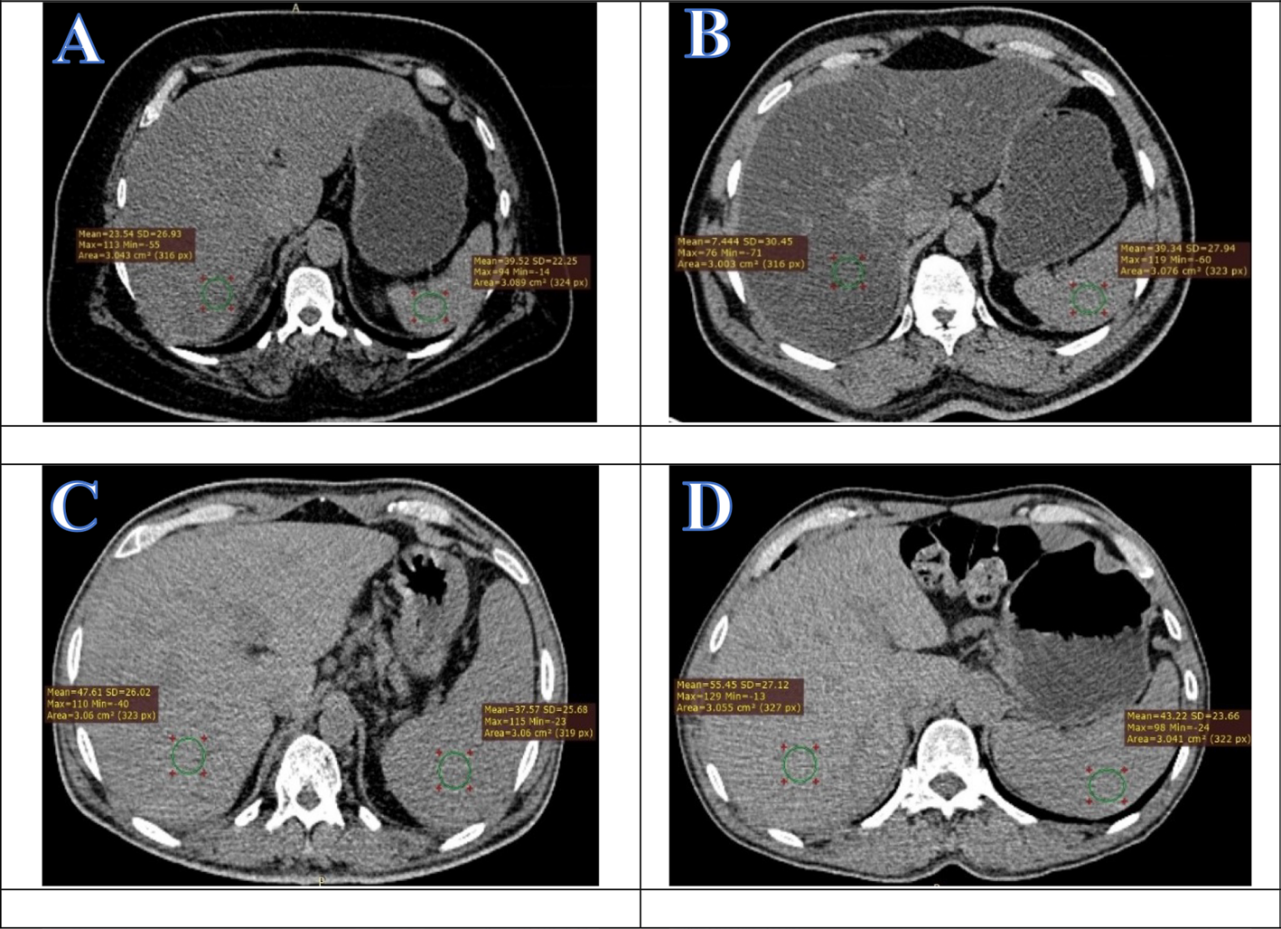
HU of the liver and spleen (mean ± SD) in cases of fatty liver (**A** & **B**) and Non-fatty liver groups (**C** & **D**). **A**: liver: 23.54 ± 26.93; spleen: 39.52 ± 22.25; **B**: liver: 7.44 ± 30.45; spleen = 39.34 ± 27.94, **C**: liver: 47.61 ± 26.02; spleen: 37.57 ± 25.68; **D**: liver: 55.45 ± 27.12; spleen: 43.22 ± 23.66

No significant difference was observed between the two groups regarding age (*p* = 0.286) or sex (*p* = 0.564), or any of the anthropometric parameters [weight (Kg; *p* = 0.104), height (cm; *p* = 0.611, BMI (Kg/m^2^; *p* = 0.055), waist circumference (cm; *p* = 0.159), body surface area (BSA: cm^2^; *p* = 0.170). About 64% and 52% of fatty liver and non-fatty liver groups were obese; respectively. Also, BSA increased in 60% and 44% in fatty liver and non-fatty liver groups; respectively. The primary tumor type varied between nineteen types of tumors in both groups. The main tumors detected in fatty liver groups were colon, Hodgkin lymphoma (HL), breast, uterus, neurodegenerative, ovarian, testicular, axillary, bone, lung and nasal tumors (from higher to lower frequency). While in nonfatty liver groups tumors were mainly HL, colon, non-Hodgkin lymphoma (NHL), axillary, breast, lung, urinary bladder (UB), Neck, thymic, and gastric tumors (from higher to lower frequency). ALT, AST, GGT, TG, and FPG were increased in 16% (4/25), 24% (6/25), 16% (4/25), 32% (8/25), and 44% (11/25) of fatty liver group and in 8% (2/25), 28% (7/25), 32% (8/25), 20% (5/25), and 12% (3/25) of nonfatty liver group; respectively. Liver enzyme ratio (AST/ALT ratio) < 1 indicates NAFLD where ALT, AST, and GGT are high (Hall & Cash, 2012). AST/ALT ratio indicated NAFLD in 64 and 56% of fatty and non-fatty liver groups; respectively. However, no significant difference was detected between fatty and non-fatty groups regarding ALT (*p *= 0.165) or AST (*p* = 0.478) or GGT (*p* = 0.620). Although diabetes was evident in each group, no significant difference was observed in FPG (*p* = 0.109) between the two groups. Despite the insignificant difference between the two groups regarding the level of TG (*p* = 0.145), the triglyceride index (TyG index) suggested insulin resistance (IR) in 96% and 80% of fatty and non-fatty liver groups; respectively. It also suggested a high likelihood of NAFLD in 92% and 76% of fatty and non-fatty liver groups. However, FLI assured fatty liver in 84% and 64% of fatty and non-fatty liver groups; respectively. NAFLD was ruled in by HSI in 88% and 64% of fatty and non-fatty liver groups; respectively. HSI significantly increased in fatty liver group compared to non-fatty liver group (*p* = 0.049). Both FLI and HSI showed a significant direct correlation with BMI in both groups (Table 1). Fibrosis-4 (FIB-4) score (Seraj et al. 2019) indicated fibrosis in (20/50) and 40% of fatty and non-fatty liver groups; respectively. Albumin level was available only for two patients in the fatty liver group and NAFLD fibrosis (NFS) score (Ahmed et al. 2022) indicated F3-F4 (advanced/sever) fibrosis in both of the two patients. All patients with high FIB-4 and NFS received either chemotherapy or radiotherapy. Hounsfield unit (HU) of liver and spleen of fatty and non- fatty liver cases are shown in Fig. 1a. A significant decrease in HU and HU liver to HU spleen ratio was observed (*p* < 0.001) of fatty liver group in comparison to their respective values in the non-fatty liver group (Table 2). While no significant difference was detected in HU of spleen in non-fatty liver group compared to fatty liver group (Table 2). The SUV in liver tissue using 6 cm^2^ ROI is shown in Fig. 1 b. (Table 3). A significant direct correlation between SUVmax and BMI was observed in non-fatty group only (Table 1). SUVmax significantly correlated with 18F–FDG dose in non-fatty group only (Table 4). While, no correlation was observed between SUV and HU liver/ HU spleen ratio (Table 5).

**Table 1:** Correlation between BMI with SUV, FLI, and HSI in the studied group

BMI (kg/m^2^) versus	Fatty liver (*n* = 25)		Non-fatty liver (*n* = 25)	
	*r*	*p*	*r*	*p*
SUV max	0.149	0.478	0.411	0.041^*^
SUV mean	0.150	0.473	0.386	0.056
FLI	0.757	< 0.001^*^	0.851	< 0.001^*^
HSI	0.882	< 0.001^*^	0.881	< 0.001^*^

r: Pearson coefficient; *: Statistically significant at *p* ≤ 0.05

**Table 2:** HU liver, HU spleen, and HU Liver/HU spleen ratio among the studied groups

Parameters	Fatty liver (*n* = 25)	Non-fatty liver (*n* = 25)	Test of Sig	*p* value
HU liver				
Min.–Max	7.0–45.0	47.0–57.0	*t* = 10.070	< 0.001^*^
Mean ± SD	29.68 ± 10.09	51.32 ± 3.68		
Median	30.0	50.0		
HU spleen				
Min.–Max	36.0–51.0	27.0–49.0	*U* = 293.50	0.711
Mean ± SD	41.80 ± 4.02	44.11 ± 5.05		
Median	41.0	42.0		
HU liver/HU spleen ratio				
Min.–Max	0.18–0.96	0.53–1.96	*U* = 19.0	< 0.001^*^
Mean ± SD	0.70 ± 0.21	1.26 ± 0.23		
Median	0.76	1.27		

U: Mann Whitney test; t: Student t-test; SD: Standard deviation; *: Statistically significant at *p* ≤ 0.05

**Table 3:** Comparison between the two studied groups according to SUV max, SUV mean using ROI of 6 cm^2^, and 18F–FDG dose

Parameters	Fatty liver (*n* = 25)	Non-fatty liver (*n* = 25)	Test of Sig	*p* value
SUV max				
Min.–Max	3.58–9.43	2.56–5.51	*U* = 33.0	< 0.001^*^
Mean ± SD	5.77 ± 1.09	3.80 ± 0.74		
Median	5.72	3.81		
SUV mean				
Min.–Max	3.29–6.81	1.97–4.76	*t* = 8.168	< 0.001^*^
Mean ± SD	4.60 ± 0.81	2.88 ± 0.68		
Median	4.37	2.92		
^18^F–FDG dose				
Min.–Max	2.10–4.50	1.80–4.20	*t* = 2.225	0.031^*^
Mean ± SD	3.37 ± 0.58	2.97 ± 0.70		
Median	3.50	2.90		

U: Mann Whitney test; t: Student t-test; SD: Standard deviation; *p*: *p* value for comparing

between the studied groups; *: Statistically significant at *p* ≤ 0.05

**Table 4:** Correlation between SUV and 18F–FDG dose in the studied groups

^18^F–FDG dose vs	Fatty liver (*n* = 25)		Non fatty liver (*n* = 25)	
	*r*	*p*	*r*	*p*
SUV max	0.336	0.100	0.413	0.040^*^
SUV mean	0.265	0.201	0.370	0.069

**Table 5:** Correlation between SUV and HU liver/ HU spleen ratio in the studied groups

HU Liver/ HU Spleen Ratio vs	Fatty liver (*n* = 25)		Non fatty liver (*n* = 25)	
	*r*	*p*	*r*	*p*
SUV max	0.124	0.556	0.020	0.924
SUV mean	0.063	0.763	0.018	0.931

r: Pearson coefficient

## Discussion

**Fig. 2:**
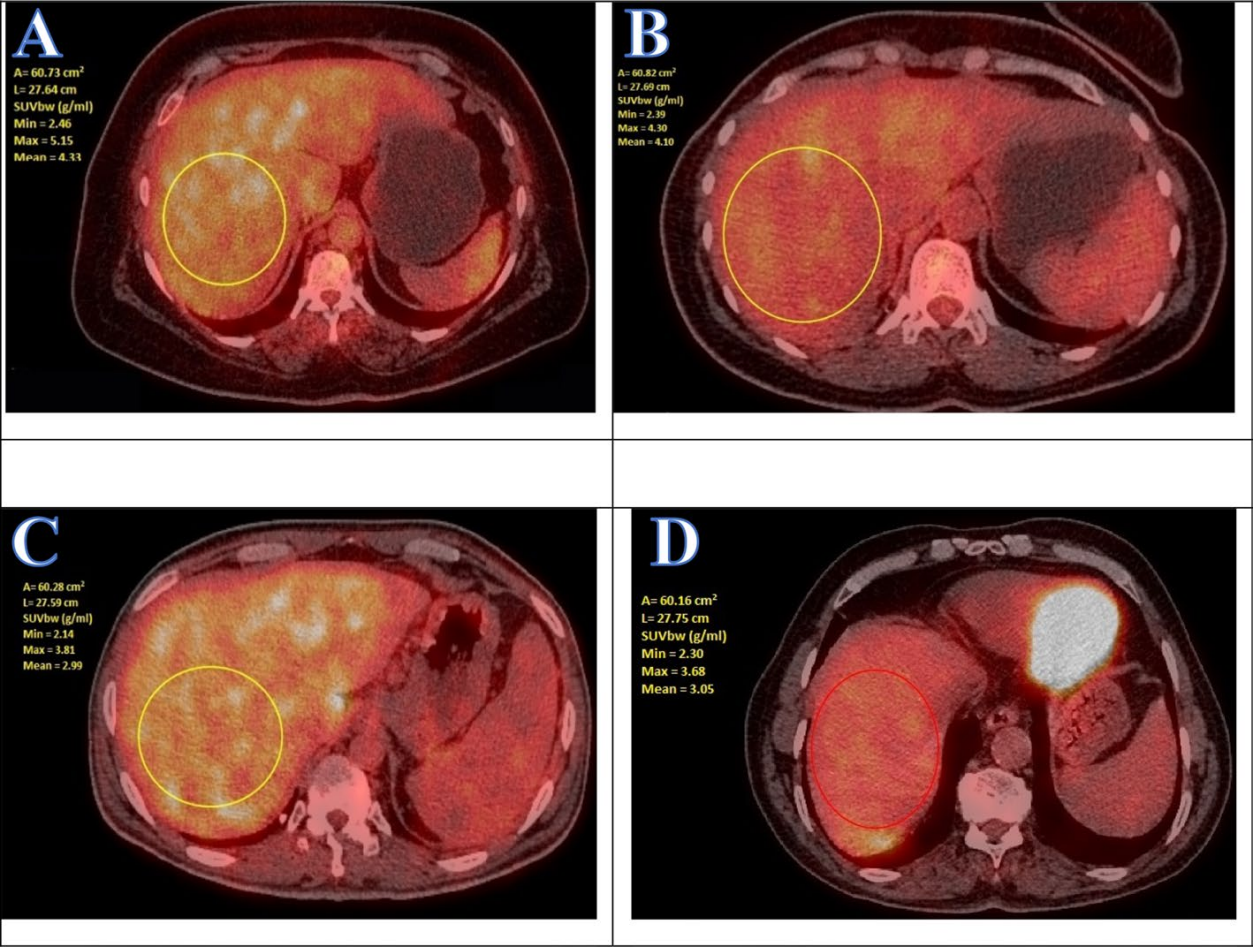
SUV of the fatty liver cases (**A** & **B**) and non-fatty liver (**C** & **D**) groups. **A**: SUV Min = 2.467, SUV Max = 5.158, Mean = 4337; **B**: SUV Min = 2.39, SUV Max = 4.30, Mean = 410. **C**: SUV Min = 2.140, SUV Max = 3.813, Mean = 2990; **D**: SUV Min = 2.307, SUV Max = 3.688, Mean = 3053

The current study provided an evaluation of liver attenuation using a liver/spleen ratio < 1 to define the prevalence of liver fat (Fig. 2). These measures are easy to obtain on CT scans where images of the liver and spleen are available. Our results emphasize that caution should be taken when liver is used as a comparator during PET-CT scan oncological studies. Hepatic steatosis causes a statistically significant increase in liver metabolic activity as measured by SUV mean and SUVmax values in fatty liver patients using CT-18F-FDG PET scan than in non-fatty liver patients. These metabolic changes might reflect an increase in the inflammatory process of the liver tissue. BMI correlated with SUV and FDG dose in non-fatty liver group and these findings require further investigations. Several imaging modalities can detect fatty liver status. The US was reported to have moderate sensitivity (65%) and specificity (81%) in detecting mild hepatic steatosis while it has good sensitivity (84.8%) and specificity (93.6%) in detecting moderate to severe hepatic steatosis (Angulo et al. 2007).

In the present study, CT was able to detect deposition of fats in 11 cases (grade zero) that were missed by the US as well as excluding fatty deposition in 11 cases (grade 1: 2; grade 2: 5; and grade 3: 2 cases) that were defied as fatty liver cases by the US. The overall agreement for US and CT has 56%. CT attenuation can be affected by tissue density, attenuation, and scanning parameters, and tiny fractions of hepatic fat may be undetected by CT” (Angulo et al. 2007; Hernaez et al. 2011). However, Steatosis can be detected if liver HU is ≤ 40 or liver HU is at least 10 less than the HU spleen (Bohte et al. 2011; Pirmoazen et al. 2020). Accordingly, steatosis was detected in 84% of the fatty liver group (21/25) where liver HU was ≤ 40) and 12 (48%) of those cases had liver HU is at least 10 less than HU spleen. In contrast, no cases were detected with these criteria among the non-fatty liver group. Further multicentral studies using various imaging modalities in comparison to the golden standard “liver biopsy” are mandatory to validate the most accurate non- invasive modality. In our study and others (Zhang et al. 2017; Kodama et al. 2007), no significant correlation was detected between age or gender and fatty liver status despite the sample size. Although Pak et al. (2012) reported that “high BMI (≥ 25) is an independent, dose-dependent risk factor for the fatty liver”, our results and Liu et al. (2015) showed no significant difference between the fatty and non-fatty liver groups regarding the weight, and height (the main components for calculating BMI). Contrary to Liu et al. (2015), we could not detect any significant difference between the fatty and non-fatty liver groups regarding the BMI although the percentage of subjects with ≥ 25 is higher in the fatty liver group than in the non-fatty liver group (80 versus 56%). This would emphasize the fact that not all patients with FLD are obese but they often have metabolic syndrome and IR as obesity-associated risk factors (Pak et al. 2012; Fan et al. 2018). Therefore, it was proposed to change the name of nonalcoholic fatty liver disease (NAFLD) to obesity-associated fatty liver disease (OAFLD) and metabolic- associated FLD (MAFLD) (Pak et al. 2012; Fan et al. 2018). Pak et al. (2012) identified three main diagnostic criteria for MAFLD clinical evidence of metabolic dysregulation; (I) overweight/obesity, (II) type 2 diabetes, and (III) clinical evidence of metabolic dysregulation. The presence of one criterion is sufficient to diagnose MAFLD. In our groups, the prevalence of obesity, type 2 diabetes, and metabolic dysregulation represented by IR (as detected by the TyG index) exceeded 50, 20, and 80%; respectively. Abdominal obesity, hypertension, dyslipidemia, and hyperglycemia were also considered among the metabolic syndrome (MS) associated with MAFLD (Softic and Kahn 2019). In our groups, abdominal obesity (as reflected by WC) showed an insignificant difference. Regardless of the higher percentage of hyperglycemia and dyslipidemia (as measured by TG) in the fatty liver group than in the non-fatty group (44 versus 12% & 32 versus 20%; respectively), no significant difference was observed as well. This would emphasize the complexity of the disease and involvements of genetic, epigenetic, environmental, metabolic factors, geographic location, the involvement of multiple organs, and various mechanisms (Alharthi and Eslam 2021). Liver enzymes (ALT, AST, and GGT) are considered indicators of liver injury. Elevated liver transaminases are common in NAFLD with evidence of metabolic syndrome [high WC, elevated blood pressure, high serum TG levels and low serum high-density lipoprotein (HDL) levels, hyperglycemia, or evidence of IR] (Alam and Fahim 2021). Juanola et al. (2021) showed a significant difference between fatty and non-fatty liver cohort regarding ALT (*p* = 0.021), AST (*p* = 0.016), GGT (*p* = 0.009), and AST/ALT ratio (*p* = 0.028). Apart from the fact that ALT, AST, GGT, and AST/ALT ratio elevations are detected in some patients of our two studied groups, no significant difference was observed. However, it is recommended to follow up with patients with elevated ALT, AST, and GGT since they correlate with the fibrosis progression in NAFLD (Oh et al. 2017; Sanyal et al. 2015; Canbakan et al. 2007). Also, the coexistence of elevated transaminases with metabolic syndrome, type 2 diabetes, hypertension, a subclinical hypothyroidism was reported recently and protection of liver function in those patients is recommended (Kleiner et al. 2019). NAFLD is divided into the nonalcoholic fatty liver (hepatic steatosis without inflammation) and nonalcoholic steatohepatitis (hepatocyte injury with ballooning of cells, inflammation, and in severe cases, fibrosis) that might progress to cirrhosis and hepatocellular carcinoma (HCC). Therefore, it is challenging to determine patients with a high risk of progression. Recent guidelines suggested a screening system for NAFLD including the use of liver function biomarkers, and variable indices (Giri et al. 2022; Jiang et al. 2021; Tokushige et al. 2021; Kang et al. 2021). AST/ ALT ratio, TyG index, FLI, and HIS reflected NAFLD in our groups with variable percentages (AST/ALT: 64 versus 56%; TyG index: 92 versus 76%; FLI: 84 versus 64%; HSI: 88 versus 64% in fatty and non-fatty liver groups; respectively). The four indices showed a weak agreement of 48% in fatty and 32% in non-fatty liver groups. FLI and HSI showed a significant direct correlation with BMI. Some of our cases in both groups showed liver fibrosis as detected using FIB-4 and NFS scores (when available). It is evident now that obesity affects various cellular responses that enhance hepatic metabolism, liver injury, and NAFLD progression while hider liver regeneration (Kitae et al. 2019). Since it was shown that chemotherapy leads to the number of cycles dependent fatty liver changes in patients with lymphoma (Oh et al. 2017), it is important to mention that all our patients with high FIB-4 and NFS received either chemotherapy or radiotherapy (patients with lymphoma were five in the fatty liver group and ten in the non-fatty liver groups). Interestingly, our results showed a significant correlation between SUVmax and each of 18F–FDG dose and BMI in nonfatty group only which may be due to the type of tumor and treatments used in this group. Thus, the link between fatty liver changes and treatment regimens in cancer therapy warrants intensive future investigations.

Although Della (2020) showed that FDG has limited access to adipose tissue, our fatty liver groups showed significant increased FDG dose than non-fatty liver group. Also, our results showed no correlation was observed between SUV and HU liver/ HU spleen ratio. A possible explanation would be that the FDG accumulated in local fat-induced micro-inflammatory foci. FDG was shown to accumulate at the sites of infection and inflammation due to increased glycolytic activity of inflammatory cells (such as neutrophils, lymphocytes, and macrophages) that use glucose as an energy source only after activation during the metabolic burst (Salama et al. 2020; Christen et al. 2010). This is supported by the fact that both SUVmax, SUV mean, and HSI significantly increased in our fatty liver group. Stumpe and Strobel (2006) also suggested that the increased SUVmax and SUV mean would be due to higher metabolic activity of liver infiltrating inflammatory cells. In the presence of hepatic inflammation, hepatic FDG uptake postulated to be increased as a result of irreversible FDG accumulation I inflammatory cells superimposed on reversible hepatocyte uptake suggesting that FDG-PET could be developed as a potential imaging approach for an early detection of NASH (Hu et al. 2022; Juanola et al. 2021; Glaudemans et al. 2013). Moreover, elevated serum GGT and TGs (as markers of hepatic inflammation and injury) shown to associate with increased hepatic glucose uptake (Bural et al. 2010). GGT elevated in 16% and 32% of our fatty and non-fatty liver groups, while TG elevated in 32% and 20% of them, respectively. Paul (2020) showed that hepatic FDG uptake is also associated with future cardiovascular and cardio-cerebrovascular events in asymptomatic individuals with NAFLD. In conclusion further corrections should be considered when the liver is used as a comparator for PET-CT scans of cancer patients. Future studies are mandatory to understand the correlation between BMI and both SUV and FDG dose in non-fatty liver cancer patients.

## Data Availability

Available when request by send an email to correspond author.

## Abbreviations

ALT: Alanine amino transferase
AST: Aspartate amino transferase
BMI: Body mass index
BSA: Body surface area
CT: Computed tomography
DICOM: Digital imaging and communications in medicine
FDG-PET/CT: Fluorodeoxyglucose positron emission tomography/CT
FIB-4 score: Fibrosis-4 score
FLI: Fatty liver index
FPG: Fasting plasma glucose
GGT: Gamma-glutamyl transferase
HCC: Hepatocellular carcinoma
HDL: High-density lipoprotein
HL: Hodgkin lymphoma
HRUS: High-resolution ultrasound
HSI: Hepatic steatosis index
HU: Hounsfield unit
IR: Insulin resistance
MAFLD: Metabolic associated fatty liver disease
MRI: Magnetic resonance imaging
MS: Metabolic syndrome
NAFLD: Non-alcoholic fatty liver disease
NASH: Non-alcoholic steatohepatitis
NCCT: Non-contrast computed tomography
NFS score: NAFLD fibrosis score
NHL: Non-Hodgkin lymphoma
OAFLD: Obesity-associated fatty liver disease
ROI: Region of interest
SUVs: Standardized uptake values
SUVmax: SUV maximum
TGs: Triglycerides
TyG index: Triglyceride index
UB: Urinary bladder
US: Ultrasonography
WC: Waist circumference
